# COVID-19: Public and patient involvement, now more than ever

**DOI:** 10.12688/hrbopenres.13067.1

**Published:** 2020-06-08

**Authors:** Edel Murphy, Edel Tierney, Éidín Ní Shé, Martha Killilea, Casey Donaghey, Anne Daly, Mary Roche, Deirdre Mac Loughlin, PPI Ignite @ NUI Galway Public Advisory Panel, Sean Dinneen

**Affiliations:** 1 PPI Ignite @ NUI Galway, NUI Galway, Galway, Ireland; 2 Public Advisory Panel, PPI Ignite @ NUI Galway, (voluntary co-facilitator), Ireland; 3 UCD School of Nursing Midwifery and Health Systems, Belfield, Dublin, Ireland; 4 Public Advisory Panel, PPI Ignite @ NUI Galway, Galway, Ireland

**Keywords:** Public and patient involvement, PPI, COVID-19

## Abstract

The research community is responding with speed to the COVID-19 pandemic, with rapid response mechanisms to fund research, shortened application turnaround times, and expedited research ethics processes. Public and patient involvement (PPI) is under pressure in this rapid response research, where it is easy for researchers and funders to dismiss PPI as non-essential, an added extra, a “nice to have”.

In this open letter, we, researchers and PPI contributors, argue that PPI is important, now more than ever. The pandemic is impacting everyone in society, with normal rules of engagement discarded. The solution to overcoming this virus will come from many different sources and many changes will emerge to healthcare delivery and to how we live our lives. It is essential that the research to find solutions is shaped by all who will be impacted: the public and the patient must be central contributors and their voice must be hear.

## Why does public and patient involvement (PPI) matter?

In recent years, there has been an increased emphasis on PPI in health and social care research ( INVOLVE, 2015; Staniszewska *et al.*, 2018). Research funders highlight the importance of PPI, increasingly requiring that research applications include PPI. Drawing on the lived experience of PPI contributors to shape the research, good quality PPI can enhance the quality and relevance of the research undertaken ( Domecq *et al.*, 2014). Moral and ethical values underpin the argument that PPI in research is an imperative: the fundamental human right to have a say and the ensuing increased public accountability and transparency, particularly in publicly funded research ( Gradinger *et al.*, 2013). In Ireland, the Health Research Board (HRB) has been at the fore of promoting PPI in research and with the Irish Research Council, in 2017 launched a joint call entitled PPI Ignite, to support higher education institutions to embed PPI into their organisational structures. The use of different PPI approaches is increasingly evident in research in Ireland ( Dwyer *et al.*, 2020; Ní Shé *et al.*, 2019; O’Hara *et al.*, 2017; O’Shea *et al.*, 2019; Tierney *et al.*, 2018; Walsh *et al.*, 2018).

It is worth reflecting on and comparing the approach to ethics in research with that taken to PPI in research. The former is established through international charters, is hard-wired into the policies and procedures of institutions and obtaining approval from a Research Ethics Committees is a necessary step to initiate most research studies. On the other hand, it is easy for researchers and funders to dismiss PPI as non-essential, an added extra, a “nice to have”. Here, drawing on formal and informal conversations with diverse PPI partners in recent weeks, we argue that PPI should be established with similar governance and structures as research ethics and be embedded in health and social care research, both during and post the COVID-19 pandemic. While we focus here on the situation in Ireland, we believe that our thoughts will also resonate with public, patients and researchers internationally.

## COVID-19 response research

The Irish research community, in common with colleagues worldwide, has rushed to respond to the COVID-19 pandemic. We have seen rapid response mechanisms to fund research, with shortened submission, review and study start-up times, and, in Ireland, the establishment of a temporary National Research Ethics Committee (NREC) to fast track COVID-19 related ethics applications. A spirit of co-operation is evident between research groups, across Universities and Hospital Groups, with the Health Services Executive and the Irish government, facilitating collaborative working, nationally and internationally. While the response from the medical and research communities has, in many ways, been inspirational with innovative new technologies emerging, some concerns are being raised in the published literature ( Glasziou *et al.*, 2020; O'Sullivan *et al.*, 2020) and elsewhere ( Kiely & Heavin, 2020), questioning the speed of the response.

## PPI under pressure in COVID-19 response research

Early signs suggest that PPI is being sidelined. The ‘expert voice’ dominates – the voice of the clinical and public health perspectives: stop transmission, find a vaccine, find a treatment, develop new ventilators. Normal rules of engagement around almost everything in society do not currently apply. We are experiencing centralised decision-making, with no time for debate and questioning. This is not a supportive environment in which the public or patient voice can be heard. In particular, research-funding calls have not emphasised the need for PPI in research proposals and there is little public review of funding applications.

PPI in research depends traditionally on personal relationships, on face-to-face meetings, on gradually building PPI capacity among both researchers and PPI partners. It is widely acknowledged that establishing these relationships takes time and commitment, from both researchers and PPI partners. In some research teams with an existing PPI ethos, re-assignment of key researchers to other roles, prioritising support for front-line activities, means that PPI skills may not be readily available. So in many cases, it has been easier to discount PPI in research during the pandemic, rather than find alternative ways to maintain existing, or build new, PPI relationships.

## PPI is important, now more than ever

We argue that in the research response to COVID-19 pandemic, PPI is important, now more than ever (a phrase used to first enshrine in health policy the concept of community participation in healthcare ( WHO, 1978; WHO, 2008). Solving the current crisis is dependent on the response of every individual in society. PPI is about researchers finding the “nuggets of gold” that come from PPI contributors. The solution to overcoming this virus will come from many different sources and the public and the patient must be central contributors and should not be silenced.

People who have experienced COVID-19, and ICU care in particular, their family members and people living with chronic conditions can draw on their lived experience to help clinicians and researchers shape and test new treatments and new approaches to care delivery. The pandemic affects everyone in society, but it does not affect everyone in the same way. The public at large, and those from minority or marginalised groups in particular, can play an important role in shaping research that explores the impact of the pandemic on our working lives, our home life and how we are coping with our “new normal”. It is important to recognise and harness the different types of knowledge and experiences brought by diverse communities and individuals: this input can help reveal the true natures of the varying experiences of the pandemic ( Marston *et al.*, 2020). Separately, public review of research applications would enhance transparency, and has the potential to bring a focus on research participant fatigue, question research duplication and waste, and objectively interrogate the potential impact of the research findings.

The Irish public embraced the initial public health campaign with its emphasis on staying at home, handwashing, respiratory etiquette and social distancing and in the words of the Taoiseach of Ireland, “ *thousands of lives have been saved*” ( www.gov.ie, 2020). Given the extent of the restrictions on normal life to fight the virus, it seems incongruous, particularly where research is publicly funded, that the public voice is excluded when planning research to find solutions and to explore the impact of the current restrictions.

Many changes will emerge from this pandemic. These changes will extend far beyond how we organise our healthcare systems; they will involve how we travel, work, educate our children and how we interact with other humans. To ensure successful adoption and adherence to these new ways of living, it is essential that solutions are shaped by those who will be most affected. For example, uptake of any new vaccines found will need the trust of the public. Involving the patient and public from the start in development of these vaccines will lead to increased transparency of the research and we have seen in recent years the positive impact of a public ambassador on the uptake of a safe and effective vaccine ( Irish Cancer Society, 2018).

### PPI essentials in a pandemic

**Figure 1.**
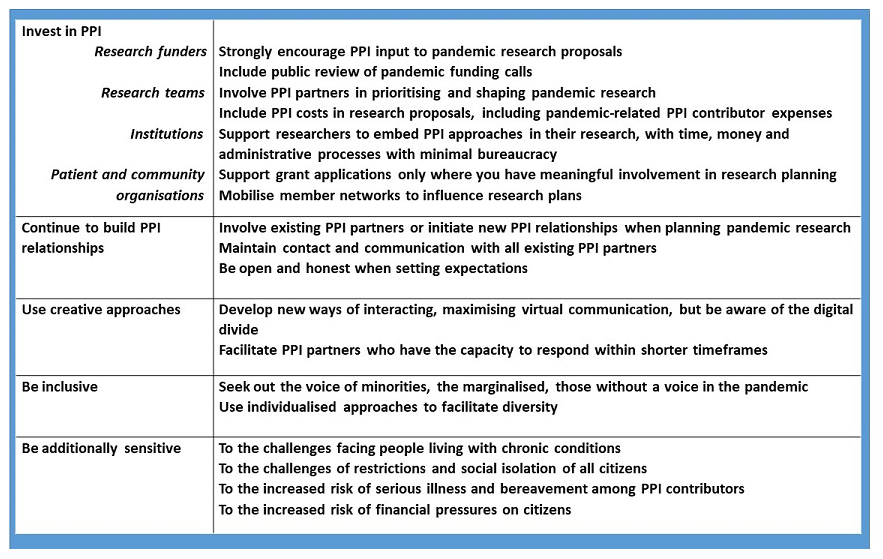
PPI essentials in a pandemic.

Now more than ever fundamental aspects of good involvement apply, but we must also find new and creative ways to ensure that the patient voice continues to be heard, both in COVID-19 rapid response research and in other research ongoing during the pandemic. Figure 1 outlines what we believe are the essential features of good involvement of public and patients in research during this pandemic. We must challenge ourselves to facilitate different formats for discussion, timings, levels of formality, and ways of communicating, tailored to the needs of different contributors, to ensure that those marginalised are represented.

## PPI responses to date

There have been some positive developments in Ireland. Some rapid response grant applications have had PPI input, while in other cases, PPI has been omitted in the COVID-19 rapid response research. Existing PPI contributors are helping other COVID-19 research teams (for example, www.iHealthfacts.ie). Some existing PPI groups have moved meetings online and other research teams are now exploring whether and how they can start new PPI initiatives. Patient and community organisations are advocating for and supporting members to move to virtual environments and to continue to work with researchers ( HRCI, 2020; IPPOSI, 2020).

The international PPI response also has been mixed, with some excellent examples of existing PPI panels adapting to cope with the “new normal”. The response in Australia, led by Anne McKenzie, to establish a national PPI panel available to support COVID-19 research ( Telathon Kids Institute, 2020) is similar to the NREC COVID-19 established in Ireland. In the UK, Health Data Research UK established a PPI group available to work with UK researchers ( HDR UK, 2020) and the Public Involvement Senior Leadership Team at the National Institute for Health Research (NIHR) has agreed new commitments for patient and public involvement, engagement and participation during the COVID-19 pandemic ( www.nihr.ac.uk, 2020).

## PPI in the pandemic: next steps

We call on all stakeholders in Ireland to take immediate steps as follows:

• **Department of Health:** establish a **National PPI Advisory Panel**, to provide the public and patient perspective to COVID-19 research plans and to increase transparency on research funding decisions, similar to the NREC COVID-19.

• **Research funders:** in all funding calls during the COVID-19 pandemic, include a public review and strongly encourage PPI in applications; be flexible in allowing funding reallocation if there was initially no provision for PPI activities in studies funded under rapid response funding calls to date.

• **Researchers:** use a mixture of existing approaches and new creative ways to ensure that PPI contributors influence all stages of your research, in spite of the changed environment for working together. It is never too late in a study to begin to involve PPI partners. Prioritise diversity and develop new approaches *with* your PPI partners, asking them what works well and what is not effective.

• **PPI advocates and patient organisations:** make your voice heard, campaign for PPI in COVID-19 research in particular, and support your members to contribute.

• **Policy makers:** ensure that a diverse public voice is heard at the policy-making table.

Everyone in society is experiencing the pandemic, but not all are impacted in the same way, with health and social inequalities very evident. Now more than ever it is an imperative that a broad and inclusive public and patient voice shapes pandemic response research, is involved in research funding decisions, is heard at the policy table and is positioned to act as an advocate for the changes to health and civil society that will undoubtedly occur. Together we are stronger.

## Data availability

No data are associated with this article.

## Author information

Members of the PPI Ignite @ NUI Galway Public Advisory Panel include Anne Daly, Casey Donaghey, Jack Gaffey, Deirdre Mac Loughlin, Helen Ogbu, Tony Regan and Mary Roche.
