# Supplementary material for: COVID-19: Public and patient involvement, now more than ever
Source: HRB Open Res. 2020 Jun 8;3:35. [Version 1] doi: 10.12688/hrbopenres.13067.1 (PMC7327728; doi:10.12688/hrbopenres.13067.1)
Supplement: Peer review history

# Peer review: Reviewer response for version 1

**DOI:** 10.21956/hrbopenres.14166.r27510

**Published:** 2020-06-30

**Authors:** Nana Folmann Hempler

**Affiliations:** 1 Health Promotion Research, Steno Diabetes Center Copenhagen, Copenhagen, Denmark

I have reviewed the manuscript titled ‘COVID-19: Public and patient involvement, now more’. The aim of letter is to argue that PPI in research is important (now more than ever) in light of the COVID-19 situation. It is very relevant and provides some very good suggestions for next steps.

 My comments are as follows:

 Abstract

- A ‘d’ is lacking in the last sentence…and their voices must be heard. 
- The letter argues that COVID-19 is more important than ever and I agree but some reflections on ‘why’ would be great and is lacking. Also, it could be noted that the letter will introduce next step or highlight the main message.
- I would suggest that the wording ‘… where it is easy for researchers and funders to dismiss to’ is changed to ‘…,where there is a high risk for researchers and funders to…’.

Main text

- I would suggest that the first section ‘why does public and patient involvement (PPI) matter?' could focus on PPI in relation to COVID-19 and less on PPI in general.
- The section ‘PPI under pressure in COVID-19 response research’ has a broad focus on PPI and COVID-19. I would suggest that it only focuses on COVID-19 research and PPI. 
- Why is PPI in COVID-19 research important? It is mentioned in the letter that solving the crisis is dependent on the response of every individual, but it would be great if this could be elaborated a bit.
- Figure 1 is great.
- COVID-19 has unearthed the deep ethnic and social inequalities that exist in societies (this could be mentioned). Also, the letter points to this very important aspect – involvement of hardly reached groups. Is it possible to give some suggestions on how to involve hardly reached groups?

Does the article adequately reference differing views and opinions?

Yes

Are all factual statements correct, and are statements and arguments made adequately supported by citations?

Yes

Is the Open Letter written in accessible language?

Yes

Where applicable, are recommendations and next steps explained clearly for others to follow?

Yes

Is the rationale for the Open Letter provided in sufficient detail?

Yes

Reviewer Expertise:

My research focuses on ethnic and social differences in the field of diabetes with a particular focus on development and implementation of interventions that improve diabetes management in hardly reached groups. In particular, I am interested in aspects that shape health behaviour change and motivation. With a background in public health, my work is positioned at the intersection of epidemiological, health services research, applied research and education research. I make use of a variety of methods, among these the design-based approach, where the target group is actively involved in the research process, which in my opinion, is where ‘the real’ innovations, in relation to improving care and support, occur.

I confirm that I have read this submission and believe that I have an appropriate level of expertise to confirm that it is of an acceptable scientific standard.

# Peer review: Reviewer response for version 1

**DOI:** 10.21956/hrbopenres.14166.r27513

**Published:** 2020-06-24

**Authors:** Steven Blackburn

**Affiliations:** 1 School of Primary, Community and Social Care, Keele University, Keele, UK; 2 NIHR Research Design Service, Keele, UK

This is a very clear and well written article on an important issue in light of the current environment.

 The authors provide compelling argument to ensure that public involvement remains central to research. As this is an open letter with an objective to persuade the research community, one might not expect inclusion of alternative viewpoints. However, justification of a few statement of facts made may be warranted. For example, there are "early signs that PPI is being sidelined" - what is this based on? The article does not provide any evidence from funders or other research institution to support this argument. It might be worth including recent concerns raised by the UK Health Research Authority about the absence of public involvement in COVID-19 research studies recently. To provide more balance, the article could be strengthened by considering some of the reasons why research teams have not undertaken public involvement, and whether these are justified. 

 Other than these minor modifications, then in my opinion, the public interest of this paper is worthy of publication.

Does the article adequately reference differing views and opinions?

No

Are all factual statements correct, and are statements and arguments made adequately supported by citations?

Partly

Is the Open Letter written in accessible language?

Yes

Where applicable, are recommendations and next steps explained clearly for others to follow?

Yes

Is the rationale for the Open Letter provided in sufficient detail?

Yes

Reviewer Expertise:

Research Fellow in Patient and Public Involvement and Engagement

I confirm that I have read this submission and believe that I have an appropriate level of expertise to confirm that it is of an acceptable scientific standard.
